# Improved Smoothed Analysis of 2-Opt for the Euclidean TSP

**DOI:** 10.1007/s00453-025-01309-9

**Published:** 2025-04-10

**Authors:** Bodo Manthey, Jesse van Rhijn

**Affiliations:** Department of Applied Mathematics, University of Twente, Enschede, The Netherlands

**Keywords:** Travelling salesperson problem, Local search, Smoothed analysis

## Abstract

The 2-opt heuristic is a simple local search heuristic for the travelling salesperson problem (TSP). Although it usually performs well in practice, its worst-case running time is exponential in the number of cities. Attempts to reconcile this difference between practice and theory have used smoothed analysis, in which adversarial instances are perturbed probabilistically. We are interested in the classical model of smoothed analysis for the Euclidean TSP, in which the perturbations are Gaussian. This model was previously used by Manthey and Veenstra, who obtained smoothed complexity bounds polynomial in *n*, the dimension *d*, and the perturbation strength $\sigma ^{-1}$. However, their analysis only works for $d \ge 4$. The only previous analysis for $d \le 3$ was performed by Englert, Röglin and Vöcking, who used a different perturbation model which can be translated to Gaussian perturbations. Their model yields bounds polynomial in *n* and $\sigma ^{-d}$, and super-exponential in *d*. As the fact that no direct analysis exists for Gaussian perturbations that yields polynomial bounds for all *d* is somewhat unsatisfactory, we perform this missing analysis. Along the way, we improve all existing smoothed complexity bounds for Euclidean 2-opt with Gaussian perturbations.

## Introduction

The Travelling Salesperson problem is a standard combinatorial optimization problem, which has attracted considerable interest from academic, educational and industrial directions. It can be stated rather compactly: given a Hamiltonian graph $G = (V, E)$ and edge weights $w: E \rightarrow \mathbb {R}$, find a minimum weight Hamiltonian cycle (tour) on *G*.

Despite this apparent simplicity, the TSP is NP-hard [10]. A particularly interesting variant of the TSP is the Euclidean TSP, in which the *n* vertices of the graph are identified with a point cloud in $\mathbb {R}^d$, and the edge weights are the Euclidean distances between these points. Even this restricted variant is NP-hard [14].

As a consequence of this hardness, practitioners often turn to heuristics. One commonly used heuristic is 2-opt [1]. This heuristic takes as its input a tour *T*, and finds two sets of two edges each, $\{e_1, e_2\} \subseteq T$ and $\{f_1, f_2\} \nsubseteq T$, such that exchanging $\{e_1, e_2\}$ for $\{f_1, f_2\}$ yields again a tour $T'$, and the total weight of $T'$ is strictly less than the total weight of *T*. This procedure is repeated with the new tour, and stops once no such edges exist. The resulting tour is said to be locally optimal.

Englert, Röglin and Vöcking constructed Euclidean TSP instances on which 2-opt can take exponentially many steps to find a locally optimal tour [8]. Despite this pessimistic result, 2-opt performs remarkably well in practice, usually requiring time sub-quadratic in *n* and obtaining tours which are only a few percent worse than the optimum [1, chapter 8].

To explain this discrepancy, the tools of probabilistic analysis have proved useful [5–8, 13]. In particular, smoothed analysis, a hybrid framework between worst-case and average-case analysis, has been successfully used in the analysis of 2-opt [7, 8, 13]. In the original version of this framework, the instances one considers are initially adversarial, and then perturbed by Gaussians. The resulting smoothed time complexity is then generally a function of the instance size *n* and the standard deviation of the Gaussian perturbations, $\sigma$.

Englert et al. obtained smoothed time complexity bounds for 2-opt on Euclidean instances by considering a more general model, in which the points are chosen in the unit hypercube according to arbitrary probability densities. The only restrictions to these densities are that (i) they are independent, and (ii) they are all bounded from above by $\phi$. Their results can be transferred to Gaussian perturbations roughly by setting $\phi = \sigma ^{-d}$, which yields a smoothed complexity that is $O(\operatorname {poly}(n, \sigma ^{-d}))$, ignoring factors depending only on *d*.

As the exponential dependence on *d* is somewhat unsatisfactory, Manthey and Veenstra [13] performed a simpler smoothed analysis yielding bounds polynomial in *n*, $1/\sigma$, and *d*. However, their analysis is limited to $d \ge 4$. While polynomial bounds for all *d* can be obtained by simply taking the result of Englert et al. for $d \in \{2, 3\}$, no smoothed analysis that directly uses Gaussian perturbations exists for these cases. We set out to perform this missing analysis, improving the smoothed complexity bounds for all $d \ge 2$ along the way.

Our analysis combines ideas from both Englert et al. and Manthey and Veenstra. From the former, we borrow the idea of conditioning on the outcomes of some of the distances between points in an arbitrary 2-change. We can then analyze the 2-change by examining the angles between certain edges in the 2-change, which are themselves random variables. From the latter, we borrow the Gaussian perturbation model (originally introduced by Spielman and Teng for the Simplex Method [15]).

We also note that in addition to improving the results of Manthey and Veenstra, our approach is significantly simpler than the analysis of Englert et al. The crux of the simplification is a carefully constructed random experiment to model a single 2-change, which allows us to bypass the need for the involved convolution integrals used by Englert et al.

We will begin by introducing some definitions and earlier results, before providing basic probability theoretical results (Sect. 2) that we will make heavy use of throughout the paper. We then proceed by analyzing a single 2-change in a similar manner as Englert et al., simplifying some of their analysis in the process (Sect. 3). Next, we prove a first smoothed complexity bound by examining so-called linked pairs of 2-changes (Sect. 4), an idea used by both Englert et al. and Manthey and Veenstra. Finally, we improve on this bound for $d \ge 3$ (Sect. 5), yielding the best known bounds for all dimensions.

## Preliminaries

### Travelling Salesperson Problem

Let $\mathcal {Y}\subseteq [-1, 1]^d$ be a point set of size *n*. The Euclidean Travelling Salesperson Problem (TSP) asks for a tour that visits each point $y \in \mathcal {Y}$ exactly once, such that the total length of the tour is minimized. The length of a tour in this variant of the TSP is the sum of the Euclidean distances between consecutive points in the tour. Formally, if the points in $\mathcal {Y}$ are visited in the order $T = (y_{\pi (i)})_{i=0}^{n-1}$ defined by a permutation $\pi$ of [*n*], then the length of the tour *T* is

$$\begin{aligned} L(T) = \sum _{i=0}^{n-1} \Vert y_{\pi (i)} - y_{\pi (i+1)}\Vert , \end{aligned}$$

where the indices are taken modulo *n*, and $\Vert \cdot \Vert$ denotes the standard Euclidean norm in $\mathbb {R}^d$. Since the Euclidean TSP is undirected, the tour $T'$ in which the vertices are visited in the reverse order has the same length as *T*. We consider these tours to be identical.

### Smoothed Analysis

Smoothed analysis is a framework for the analysis of algorithms, which was introduced in 2004 by Spielman and Teng [15]. The method is particularly suitable to algorithms with a fragile worst-case input [11]. Since its introduction, the method has been applied to a wide variety of algorithms [12, 16].

Heuristically, one imagines that an adversary chooses an input to the algorithm. The input is then perturbed in a probabilistic fashion. The hope is that any particularly pathological instances that the adversary might choose are destroyed by the random perturbation. One then computes a bound on the expected number of steps that the algorithm performs, where the expectation is taken with respect to the perturbation.

For our model of a smoothed TSP instance, we allow the adversary to choose a point set $\mathcal {Y}\subseteq [-1, 1]^d$ of size *n*. We then perturb each point $y_i \in \mathcal {Y}$ with an independent *d*-dimensional Gaussian random variable $g_i$, $i \in [n]$, with mean 0 and standard deviation $\sigma$. This yields a new point set, $\mathcal {X}= \{y_i + g_i \mid y_i \in \mathcal {Y}\}$. We will bound the expected number of steps taken by the 2-opt heuristic on the TSP instance defined by $\mathcal {X}$, with the expectation taken over this Gaussian perturbation. We will refer to this quantity as the smoothed complexity of 2-opt.

For the purposes of our analysis, we always assume that $\sigma \le 1$. This is a mild restriction, as the bound for $\sigma =1$ also applies to all larger values of $\sigma$, and small perturbations are particularly interesting in smoothed analysis.

For a general outline of the strategy, consider a 2-change where the edges $\{a, z_1\}$ and $\{b, z_2\}$ are replaced by $\{a, z_2\}$ and $\{b, z_1\}$. The change in tour length of this 2-change is

$$\begin{aligned} \Delta = \Vert a - z_1\Vert + \Vert b - z_2\Vert - \Vert a - z_2\Vert - \Vert b - z_1\Vert . \end{aligned}$$

Since the locations of the points $\{a, b, z_1, z_2\}$ are random variables, so is $\Delta$. We seek to bound the probability that there exists a 2-change whose improvement is exceedingly small, enabling us to use a potential argument.

Let $\Delta _\textrm{min}$ denote the improvement of the least-improving 2-change in the instance. If $\mathbb {P}(\Delta _\textrm{min}\le \epsilon )$ is suitably small for small $\epsilon$, then each iteration is likely to decrease the tour length by a large amount. As long as the initial tour has bounded length, this then provides a limit to the number of iterations that the heuristic can perform, since the tour length is bounded from below by 0.

### Basic Results

We state some general results that we will need at points throughout the paper.

The next lemma provides a simple framework that we can use to prove smoothed complexity bounds for 2-opt.

Let $\Delta _\textrm{min}$ denote the smallest improvement of any 2-change, and let $\Delta ^\textrm{link}_\textrm{min}$ denote the smallest improvement of any pair of linked 2-changes (see Sect. 4 for a definition of linked pairs).

#### Lemma 1

[13, Lemma 2.2] Suppose that the longest tour has a length of at most *L* with probability at least $1 - 1/n!$. Let $\alpha > 1$ be a constant. If for all $\epsilon > 0$ it holds that $\mathbb {P}(\Delta _\textrm{min} \in (0, \epsilon ]) = O\left( P\epsilon ^\alpha \right)$, then the smoothed complexity of 2-opt is bounded from above by $O(P^{1/\alpha } L)$. The same holds if we replace $\Delta _\textrm{min}$ by $\Delta _\textrm{min}^\textrm{link}$, provided that $P^{1/\alpha }L = \Omega (n^2)$.

#### Probability Theory

We provide some basic probability theoretical results. Throughout the paper, given a random variable *X*, we denote its probability density by $f_{X}$ and its cumulative distribution function by $F_X$. If we furthermore condition on some event *Y*, we write $f_{X|Y}$ for the conditional density of *X* given *Y*.

#### Chi Distributions

Suppose we are given two points $y_1, y_2 \in \mathcal {Y}$ and perturb both points with independent Gaussian random variables $g_1$ and $g_2$, resulting in $x_i = y_i + g_i$, $i \in [2]$. Then the distance $\Vert x_1 - x_2\Vert$ between the two perturbed points is distributed according to a noncentral *d*-dimensional chi distribution with noncentrality parameter $s = \Vert y_1 - y_2\Vert$, which we denote $\chi _d^s$. We call $\chi _d^0$ a central *d*-dimensional $\chi$ distribution. We have two useful expressions for the chi distribution [9]:

1 $$\begin{aligned} \chi _d^s(r) = \frac{e^{-\frac{r^2+s^2}{2\sigma ^2}} \cdot \frac{r^{d-1}}{\sigma ^d}}{(rs/\sigma ^2)^{d/2-1}} I_{d/2 - 1}\left( \frac{rs}{\sigma ^2}\right) = e^{-\frac{s^2}{2\sigma ^2}} \sum _{i=0}^\infty \frac{1}{i!}\left( \frac{s^2}{2\sigma ^2}\right) ^i \chi _{d + 2i}(r), \end{aligned}$$

where $\chi _d(r) = \chi _d^0(r)$, the central chi distribution. Here, $I_\nu (x)$ denotes the modified Bessel function of the first kind, of order $\nu > -1/2$, defined as [2]

2 $$\begin{aligned} I_\nu (x) = \sum _{k=0}^\infty \frac{1}{k!\Gamma (k + \nu + 1)} \left( \frac{x}{2} \right) ^{2k + \nu }. \end{aligned}$$

#### General Results

In the following, we use the notion of stochastic dominance. Let *X* and *Y* be two real-valued random variables. We say that *X* stochastically dominates *Y* if for all *x*, it holds that $\mathbb {P}(X \ge x) \ge \mathbb {P}(Y \ge x)$, and this inequality is strict for some *x*. We may equivalently say that the density of *X* stochastically dominates the density of *Y*.

To use Lemma 1, we need to limit the probability that any TSP tour in our smoothed instance is too long. This was previously done by Manthey and Veenstra; we state their result in Lemma 2.

##### Lemma 2

[13, Lemma 2.3] Let $c \ge 2$ be a sufficiently large constant, and let $D = c \cdot (1 + \sigma \sqrt{n \log n})$. Then $\mathbb {P}(\mathcal {X}\nsubseteq [-D, D]^d) \le 1/n!$.

The next lemma is a reformulation of another result by Manthey and Veenstra [13]. The lemma is very useful in conjunction with Lemma 4, as we will have cause to condition on the outcome of drawing noncentral *d*-dimensional chi random variables.

##### Lemma 3

[13, Lemma 2.8] The noncentral *d*-dimensional chi distribution with parameter $\mu > 0$ and standard deviation $\sigma$ stochastically dominates the central *d*-dimensional chi distribution with the same standard deviation.

The following lemma from Manthey and Veenstra is slightly generalized compared to its original statement. We do not provide a proof, since the original proof remains valid when simply replacing the original assumption with ours.

##### Lemma 4

[13, Lemma 2.7] Assume $c \in \mathbb {R}_{\ge 0}$ is a fixed constant and $d \in \mathbb {N}$ is fixed and arbitrary with $d > c$. Let $\chi _d$ denote the *d*-dimensional chi distribution with variance $\sigma ^2$. Then

$$\begin{aligned} \int _0^\infty \chi _d(x) x^{-c} \textrm{d}x = \Theta \left( \frac{1}{d^{c/2}\sigma ^c} \right) . \end{aligned}$$

### Limiting the Adversary

**Fig. 1:**
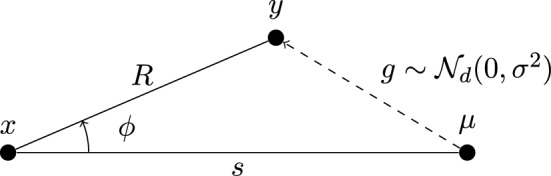
The setting of Theorem 5. As mentioned in the proof of Theorem 5, we may assume without loss of generality that $\mu$ lies on *L*

In our analysis we will closely study the angles between edges in the smoothed TSP instance. These angles can be initially specified to our detriment by the adversary. However, the power of the adversary is limited by the strength of the Gaussian perturbations. We quantify the power of the adversary in Theorem 5. See Fig. 1 for a sketch accompanying the theorem.

#### Theorem 5

Let *L* be some line in $\mathbb {R}^d$, and let $x \in L$. Let *y* be a point drawn from a *d*-dimensional Gaussian distribution with mean $\mu \in \mathbb {R}^d$ and variance $\sigma ^2$. Let $\phi$ denote the angle between *L* and $x - y$, and let $R = \Vert x-y\Vert$ and $s = \Vert x - \mu \Vert$. Let $f_{\phi |R=r}$ denote the density of $\phi$, conditioned on a specific outcome $r > 0$ for *R*. Then for all $d \ge 2$,

$$\begin{aligned} \sup _{\phi \in [0, \pi ]} f_{\phi |R=r}(\phi ) = O\left( \sqrt{d} + \frac{\sqrt{rs}}{\sigma }\right) . \end{aligned}$$

Moreover, for $$d \ge 3$$,

$$\begin{aligned} \sup _{\phi \in (0, \pi )} \frac{f_{\phi |R=r}(\phi )}{\sin \phi } = O\left( \sqrt{d} + \frac{rs}{\sigma ^2\sqrt{d}}\right) . \end{aligned}$$

Theorem 5 yields the following corollary, which provides information on the angle between two Gaussian random points in $\mathbb {R}^d$ with respect to some third point. This corollary is especially useful when analyzing 2-changes in smoothed TSP instances.

#### Corollary 6

Let $x \in \mathbb {R}^d$. Let *y* and *z* be drawn from *d*-dimensional Gaussian distributions with arbitrary means and the same variance $\sigma ^2$. Let $\phi$ denote the angle between $y - x$ and $z - x$, and let $R = \Vert x - y\Vert$ and $S = \Vert x - z\Vert$. Let $f_{\phi |R=r,S=s}$ denote the density of $\phi$ conditioned on some outcome $r > 0$ for *R* and $s > 0$ for *S*. Then for all $d \ge 2$,

$$\begin{aligned} \sup _{\phi \in [0, \pi ]} f_{\phi |R=r,S=s}(\phi ) = O\left( \sqrt{d} + \frac{\sqrt{\min \{r\bar{r}, s\bar{s}}\}}{\sigma }\right) , \end{aligned}$$

where $\bar{r} = \Vert x - \mathbb {E}(y)\Vert$ and $\bar{s} = \Vert x - \mathbb {E}(z)\Vert$. Moreover, for $d \ge 3$,

$$\begin{aligned} \sup _{\phi \in (0, \pi )} \frac{f_{\phi |R=r,S=s}(\phi )}{\sin \phi } = O\left( \sqrt{d} + \frac{\min \{r\bar{r}, s\bar{s}\}}{\sigma ^2\sqrt{d}}\right) . \end{aligned}$$

#### Proof (assuming Theorem 5)

We denote the density of $\phi$ conditioned on $R = r$ and $S = s$ by $f_{\phi |R=r,S=s}$. We perform a random experiment as follows.

If $r \le s$, then we let an adversary determine the position of *z*, subject to $S = s$. Subsequently, we draw the line *L* through *x* and *z*. Theorem 5 then yields a bound for $f_{\phi |R=r,S=s}$ of $O(\sqrt{d} + \sqrt{r\bar{r}}/\sigma )$. The same process yields the bound for $f_{\phi |R=r,S=s}(\phi )/\sin \phi$ when $d \ge 3$.

If $s \le r$, then we use a similar argument, just swapping the roles of *y* and *z*. This yields $O(\sqrt{d} + \sqrt{s\bar{s}}/\sigma )$.

Combining these two bounds yields the corollary. $\square$

The remainder of this section is devoted to proving Theorem 5. Recall the formulas for $\chi _d^s$, cf. Eq. (1). During the proof of Theorem 5, we will need to bound $\chi _d^s$ from below, for which We require some lower bounds on $I_\nu$. We thus spend some time in this section proving such bounds.

The following bound on $I_\nu$ holds for all $x \ge 0$ and $\nu > -1/2$; it results from keeping only the $k = 0$ term in Eq. (2).

#### Lemma 7

For all $x \ge 0$ and $\nu >-1/2$,

$$\begin{aligned} I_\nu (x) \ge \frac{(x/2)^\nu }{\Gamma \left( \nu + 1\right) }. \end{aligned}$$

As will become apparent during the proof of Theorem 5, the bound in Lemma 7 is too weak for large values of *x*. We thus need a stronger bound for this regime.

#### Lemma 8

Given $x > 1$ and $\nu \ge 0$, it holds that

$$\begin{aligned} I_\nu (x) \ge c_\nu \cdot \frac{e^x}{\sqrt{x}}, \end{aligned}$$

for some $c_\nu > 0$ that depends only on $\nu$.

#### Proof

First, suppose $\nu \ge 1/2$. Our starting point is the following integral representation of $I_\nu$, which holds for $\nu > -1/2$ [2]:

3 $$\begin{aligned} I_\nu (x) = \frac{(x/2)^\nu }{\pi ^{1/2}\Gamma (\nu +1/2)} \int _{-1}^1 e^{xt}(1-t^2)^{\nu -\frac{1}{2}}\textrm{d}t. \end{aligned}$$

Observe first that the factor in front of the integral is non-negative, as is the integrand. We first restrict the domain of integration to $(1-1/x, 1)$, which is permissible as $x > 1$. Next, we use the identity $(1-t^2) = (1-t)(1+t)$ to replace $(1-t^2)^{\nu -1/2}$ in the integrand by $(1-t)^{\nu -1/2}$. This yields a lower bound, since *t* only takes positive values over the restricted domain of integration, and $\nu \ge 1/2$.

Next, we substitute $u = 1-t$, which yields

$$\begin{aligned} \int _0^{1/x} e^{x(1-u)}u^{\nu -\frac{1}{2}}\textrm{d}u = e^x \int _{0}^{1/x} e^{-xu}u^{\nu -\frac{1}{2}} \textrm{d}u \ge e^x \int _0^{1/x} (1-xu)u^{\nu -\frac{1}{2}} \textrm{d}u, \end{aligned}$$

making use of the standard inequality $e^x \ge 1 + x$. Note that the integrand remains non-negative for all values of *u* over which we integrate. The remaining integral evaluates to

$$\begin{aligned} \int _0^{1/x} (1-xu)u^{\nu -\frac{1}{2}} \textrm{d}u&= \frac{1}{\nu +1/2} \frac{1}{x^{\nu +1/2}} - \frac{x}{\nu +3/2}\frac{1}{x^{\nu +3/2}} \\&= \left( \frac{1}{\nu + 1/2} - \frac{1}{\nu + 3/2}\right) x^{-\nu -1/2}. \end{aligned}$$

Thus, we are left with

$$\begin{aligned} I_\nu (x) \ge \left( \frac{1}{\nu + 1/2} - \frac{1}{\nu + 3/2}\right) \frac{1}{2^\nu \sqrt{\pi }\Gamma (\nu + 1/2)} \frac{e^x}{\sqrt{x}}. \end{aligned}$$

Letting $c_\nu$ be the entire prefactor of $e^x/\sqrt{x}$, we are done for $\nu \ge 1/2$.

The case $\nu < 1/2$ can be carried out analogously; however, rather than using $1-t^2 = (1+t)(1-t) \ge 1 - t$, we instead use $1-t^2 = (1+t)(1-t) \le 2(1-t)$, since $1-t^2$ now appears in the denominator of the integrand in Eq. (3). $\square$

While Lemma 8 is useful for large values of *x* and constant $\nu$, it is too weak for large values of $\nu$ due to the constant $c_\nu$. We can however use it to obtain another bound, which we will use at a key step in the proof of Theorem 5. First, we need the following lemma, which can be found as an equation in a paper by Amos.

#### Lemma 9

[3] For all $x > 0$ and $\nu \ge 1$,

$$\begin{aligned} \frac{I_{\nu }(x)}{I_{\nu -1}(x)} \ge \frac{\sqrt{x^2 + \nu ^2} - \nu }{x}. \end{aligned}$$

We can use this lemma recursively to bound $I_\nu$ from below for all $\nu \ge 0$, with the base case given by Lemma 8.

#### Lemma 10

There exists a constant $c > 0$ such that, for all $x > 1$ and $\nu \ge 0$,

$$\begin{aligned} I_\nu (x) \ge c\cdot \left( \frac{\sqrt{x^2 + \nu ^2} - \nu }{x}\right) ^{\nu +\frac{1}{2}} \frac{e^{\sqrt{x^2 + \nu ^2}}}{\sqrt{x}}. \end{aligned}$$

#### Proof

First, we assume $\nu \in \mathbb {N}$ for the sake of clarity; fractional $\nu$ and $\nu < 1$ will be addressed at the end of the proof. We start by using Lemma 9. Applied iteratively, it yields

$$\begin{aligned} I_\nu (x) \ge I_0(x) \prod _{k=1}^\nu \frac{\sqrt{x^2 + k^2} - k}{x} = x^\nu I_0(x) \prod _{k=1}^\nu \frac{1}{\sqrt{x^2 + k^2} + k}. \end{aligned}$$

Equivalently,

$$\begin{aligned} \frac{I_0(x) \cdot x^\nu }{I_\nu (x)} \le \prod _{k=1}^\nu \left( \sqrt{x^2 + k^2} + k\right) . \end{aligned}$$

To bound this product, we first take its logarithm to convert it to a sum:

$$\begin{aligned} \ln \prod _{k=1}^\nu \left( \sqrt{x^2 + k^2} + k\right) = \sum _{k=1}^\nu \ln \left( \sqrt{x^2 + k^2} + k\right) . \end{aligned}$$

It is tempting to now bound this sum by integrating the summand over $[1, \nu + 1]$, as the summand is monotone increasing in *k*. However, the resulting bound turns out to be slightly too weak for our purposes. Instead, we refine this by using the Euler-Maclaurin formula [4]. The formula states that, for a function *f* that is *p*-times continuously differentiable on [*m*, *n*],

$$\begin{aligned} \sum _{i=m}^n f(i) = \int _m^n f(k)\textrm{d}k + \frac{f(n) + f(m)}{2} + \sum _{k=1}^{\lfloor p/2 \rfloor } \frac{B_{2k}}{(2k)!} \left( f^{(2k-1)}(n) - f^{(2k-1)}(m)\right) + R_p, \end{aligned}$$

where $B_k$ denotes the *k*th Bernoulli number with $B_1 = \frac{1}{2}$, and $R_p$ is a remainder term. The remainder can be bounded from above as [2]

$$\begin{aligned} |R_p| \le \frac{2\zeta (p)}{(2\pi )^p}\int _m^n |f^{(p)}(x)|\textrm{d}x, \end{aligned}$$

with $\zeta$ the Riemann zeta function. We apply this formula to $f(k) = \ln (\sqrt{x^2 + k^2} + k)$. It suffices to take $p = 2$, so that we retain only the first term of the sum. We have

$$\begin{aligned} f'(k) = \frac{1}{\sqrt{x^2 + k^2}}. \end{aligned}$$

Observe that $f''(k) \le 0$ for all $x, k \in \mathbb {R}$, so we have $|f''(k)| = -f''(k)$. This enables us to write the estimate for the remainder term as

$$\begin{aligned} |R_2| \le -\frac{2\zeta (2)}{4\pi ^2} \int _1^\nu f''(k)\textrm{d}k = - \frac{1}{12} \left( f'(\nu ) - f'(1)\right) . \end{aligned}$$

Since $B_{2} = \frac{1}{6}$ [2], we obtain

$$\begin{aligned} \sum _{k=1}^\nu f(k)&= \int _1^\nu f(k) \textrm{d}k + \frac{f(1) + f(\nu )}{2} + \frac{1}{12}(f'(\nu ) - f'(1)) + R_p \\&\le \int _1^\nu f(k) \textrm{d}k + \frac{f(1) + f(\nu )}{2} + \frac{1}{6}\left| f'(\nu ) - f'(1) \right| . \end{aligned}$$

The integral evaluates to

$$\begin{aligned} \sqrt{1+x^2} - \sqrt{x^2 + \nu ^2} + \ln \left( \frac{1}{1 + \sqrt{1 + x^2}} \right) + \nu \ln \left( \sqrt{x^2 + \nu ^2} + \nu \right) . \end{aligned}$$

Meanwhile, we have

$$\begin{aligned} \frac{f(1) + f(\nu )}{2} = \ln \sqrt{1 + \sqrt{1 + x^2}} + \frac{1}{2}\ln \left( \sqrt{x^2 + \nu ^2} + \nu \right) , \end{aligned}$$

and

$$\begin{aligned} \left| f'(1) - f'(\nu ) \right| = \frac{1}{\sqrt{x^2 + 1}} - \frac{1}{\sqrt{x^2 + \nu ^2}} \le 1. \end{aligned}$$

Putting this all together,

$$\begin{aligned} \sum _{k=1}^\nu \ln (\sqrt{x^2 + \nu ^2} + \nu ) \le \sqrt{1+x^2} - \sqrt{x^2 + \nu ^2} + \ln \left( \frac{(\sqrt{x^2+\nu ^2}+\nu )^{\nu +\frac{1}{2}}}{\sqrt{1 + \sqrt{1 + x^2}}}\right) + 1. \end{aligned}$$

Exponentiating, we find

$$\begin{aligned} \frac{I_0(x)x^\nu }{I_\nu (x)} \le e\cdot \frac{e^{\sqrt{1+x^2} - \sqrt{x^2 + \nu ^2}}}{\sqrt{1 + \sqrt{1 + x^2}}} \left( \sqrt{x^2 + \nu ^2} + \nu \right) ^{\nu +\frac{1}{2}}. \end{aligned}$$

Using that $$1 + \sqrt{1 + x^2} \ge x$$,

$$\begin{aligned} I_{\nu }(x)&\ge \frac{1}{e}\cdot \left( \frac{x}{\sqrt{x^2 + \nu ^2} + \nu } \right) ^{\nu + \frac{1}{2}} e^{\sqrt{x^2 + \nu ^2} - \sqrt{1+x^2}} I_0(x) \\&= \frac{1}{e} \cdot \left( \frac{\sqrt{x^2 + \nu ^2} - \nu }{x}\right) ^{\nu +\frac{1}{2}} e^{\sqrt{x^2 + \nu ^2} - \sqrt{1+x^2}} I_0(x). \end{aligned}$$

To conclude the proof for integral $\nu$, we apply Lemma 8 for $\nu = 0$ to obtain $I_0(x) \ge c_0 \cdot e^x /\sqrt{x}$, and observe that $|\sqrt{1 + x^2} - x| \le 1$ for all $x \ge 0$.

For fractional $\nu$, one can follow the same proof, simply replacing $I_0$ by $I_{\nu '}$ for some $\nu ' \in (0, 1)$ throughout. Meanwhile, for $\nu < 1$, one can choose a suitable constant to match the bound from the lemma statement to the bound from Lemma 8. $\square$

The final piece of preparation for Theorem 5 is now the following inequality.

#### Lemma 11

Let $x \ge 0$ and $y \ge 1$. Then

$$\begin{aligned} \left( \frac{\sqrt{x^2 + y^2} + y}{\sqrt{x^2 + \left( y-\frac{1}{2}\right) ^2} + \left( y - \frac{1}{2}\right) }\right) ^y \le e. \end{aligned}$$

#### Proof

Let *f*(*x*, *y*) denote the function in brackets. We first show that *f* is nonincreasing in *x*. Observe that *f*(*x*, *y*) is nonincreasing if and only if $\ln f(x,y)$ is nonincreasing. We have

$$\begin{aligned} \frac{\partial }{\partial x} \ln \left( \sqrt{x^2+y^2} + y\right) = \frac{1}{\sqrt{x^2 + y^2} + y} \cdot \frac{x}{\sqrt{x^2+y^2}}. \end{aligned}$$

Thus,

$$\begin{aligned}&\frac{\partial }{\partial x} \ln f(x,y)= y \cdot x \\&\quad \times \left( \frac{1}{\sqrt{x^2+y^2} \left( \sqrt{x^2+y^2} + y^2\right) } - \frac{1}{\sqrt{x^2 + \left( y-\frac{1}{2}\right) ^2} \left( \sqrt{x^2 + \left( y-\frac{1}{2}\right) ^2} + \left( y-\frac{1}{2}\right) \right) } \right) . \end{aligned}$$

As the factor inside the parentheses is nonpositive and we assume $x \ge 0$ and $y \ge 1$, we see that $\ln f(x,y)$, and hence *f*(*x*, *y*), is nonincreasing in *x*.

We desire an upper bound for $f(x,y)^y$, so we set $x = 0$:

$$\begin{aligned} f(x,y)^y \le f(0,y)^y = \left( \frac{y}{y-\frac{1}{2}}\right) ^y = \left( \frac{1}{1 - \frac{1}{2y}}\right) ^y \le \left( 1 + \frac{1}{y}\right) ^y \le e, \end{aligned}$$

where the penultimate inequality holds for $y \ge 1$. $\square$

We can now prove Theorem 5.

#### Proof of Theorem 5

Observe that the upper bound on the density of $\phi$ is independent of the orientation of the line *L*. Hence, we rotate *L* about *x* such that *L* passes through $\mu$. We begin by proving the first part of the theorem.

Let $f_Y$ denote the density of *y*,

$$\begin{aligned} f_Y(y) = \frac{1}{(2\pi )^{d/2}\sigma ^d}e^{-\frac{\Vert y-\mu \Vert ^2}{2\sigma ^2}}. \end{aligned}$$

We center our coordinate system on *x*, and orient the $y_1$-axis along $\mu - x$, so that $\mu = (s, 0, \ldots , 0)$. We then switch to spherical coordinates $(r, \phi , \theta _1, \ldots , \theta _{d-2})$, where

$$\begin{aligned} y_1&= r \cos \phi , \\ y_2&= r\sin \phi \cos \theta _1, \\ y_3&= r\sin \phi \sin \theta _2\cos \theta _2, \\&\vdots \\ y_d&= r\sin \phi \sin \theta _2 \ldots \sin \theta _{d-3}\sin \theta _{d-2}. \end{aligned}$$

Here, *r* ranges from 0 to $\infty$, $\theta _{d-2}$ ranges from 0 to $2\pi$, while all other angles range from 0 to $\pi$. Due to the orientation of our coordinate system, the coordinate angle $\phi$ corresponds to the random variable $\phi$ from the theorem statement.

To compute the density of $\phi$ conditioned on $R = r$, we write

$$\begin{aligned} f_{\phi |R=r}(\phi ) = \frac{f_{\phi ,R}(\phi , r)}{f_R(r)}, \end{aligned}$$

where $f_{\phi ,R}$ denotes the joint density of $\phi$ and *R*. We obtain this density by integrating the density of $f_Y$ transformed to spherical coordinates over $\theta _1$ through $\theta _{d-2}$. Meanwhile, $f_R$ denotes the density of *R*, which is a noncentral *d*-dimensional chi distributed random variable with parameter *s*.

The joint density $f_{\phi ,R}$ is

$$\begin{aligned} f_{\phi ,R}(\phi , r) = \frac{1}{(2\pi )^{d/2}} \frac{r^{d-1}}{\sigma ^d} e^{-\frac{r^2 + \sigma ^2}{2\sigma ^2}} e^{\frac{rs\cos \phi }{\sigma ^2}} \sin ^{d-2}\phi \int _0^{2\pi }\textrm{d}\theta \prod _{k=1}^{d-3}\int _{0}^\pi \sin ^k \theta \textrm{d}\theta . \end{aligned}$$

It holds that, for $k \in \mathbb {N}$,

$$\begin{aligned} \int _0^\pi \sin ^k \theta \textrm{d}\theta = \frac{\sqrt{\pi }\Gamma \left( \frac{k+1}{2}\right) }{\Gamma \left( \frac{k+2}{2}\right) }. \end{aligned}$$

By telescoping, it follows that

$$\begin{aligned} \prod _{k=1}^{d-3} \int _0^\pi \sin ^k \theta \textrm{d}\theta = \pi ^{\frac{d-3}{2}} \cdot \frac{\Gamma (1)}{\Gamma \left( \frac{d-1}{2}\right) } = \frac{\pi ^{\frac{d-3}{2}}}{\Gamma \left( \frac{d-1}{2}\right) }. \end{aligned}$$

Inserting this into our expression for $f_{\phi ,R}$, we obtain

$$\begin{aligned} f_{\phi ,R}(\phi , r) \le \frac{2^{1-\frac{d}{2}}}{\sqrt{\pi }} \frac{r^{d-1}}{\sigma ^d}\frac{\sin ^{d-2}\phi }{\Gamma \left( \frac{d-1}{2}\right) } e^{-\frac{r^2+s^2}{2\sigma ^2}}e^{\frac{rs\cos \phi }{\sigma ^2}}. \end{aligned}$$

Next, we use the expression for $f_R$ given in Eq. (1). Combining this with the above bound for $f_{\phi ,R}$, we have

$$\begin{aligned} f_{\phi |R=r}(\phi ) \le \frac{2^{1-\frac{d}{2}}}{\sqrt{\pi }} \frac{\sin ^{d-2}\phi }{\Gamma \left( \frac{d-1}{2}\right) } \left( \frac{rs}{\sigma ^2}\right) ^{\frac{d}{2}-1} \frac{e^{\frac{rs\cos \phi }{\sigma ^2}}}{I_{d/2-1}(rs/\sigma ^2)}. \end{aligned}$$

For brevity, let $x:= rs/\sigma ^2$, and let $\nu := d/2 - 1$. Then, up to a constant, $f_{\phi |R=r}$ is bounded from above by

4 $$\begin{aligned} \frac{x^\nu \sin ^{2\nu }(\phi ) e^{x\cos \phi }}{2^\nu \Gamma \left( \nu + \frac{1}{2}\right) I_\nu (x)}. \end{aligned}$$

For any fixed *x* and $\nu$, Eq. (4) is maximized when $\phi = \phi ^*$, where $\phi ^*$ satisfies

5 $$\begin{aligned} \sin ^2 \phi ^* = \frac{2\nu }{x} \cos \phi ^*. \end{aligned}$$

Obtaining this is a matter of ordinary calculus. This equation has a unique solution in $[0, \pi ]$ of

6 $$\begin{aligned} \phi ^* = 2\arctan \left( \sqrt{\frac{\sqrt{\nu ^2 + x^2} - x}{\nu }} \right) = 2\arctan \left( \sqrt{\sqrt{x^2/\nu ^2 + 1} - x/\nu } \right) . \end{aligned}$$

It can also be verified that

7 $$\begin{aligned} \cos \phi ^* = \sqrt{1 + \frac{\nu ^2}{x^2}} - \frac{\nu }{x} = \frac{\sqrt{x^2 + \nu ^2} - \nu }{x}. \end{aligned}$$

Using this identity together with Eq. (5) in Eq. (4), we find

$$\begin{aligned} f_{\phi |R=r}(\phi )&\le \Theta (1) \cdot \frac{\nu ^\nu }{\Gamma \left( \nu +\frac{1}{2}\right) } \cdot \left( \frac{\sqrt{x^2 + \nu ^2} - \nu }{x}\right) ^\nu \cdot \frac{e^{x\left( \sqrt{1 + \frac{\nu ^2}{x^2}} - \frac{\nu }{x}\right) }}{I_\nu (x)} \\&= \Theta (1) \cdot \frac{(\nu /e)^\nu }{\Gamma \left( \nu + \frac{1}{2}\right) } \cdot \left( \frac{\sqrt{x^2 + \nu ^2} - \nu }{x}\right) ^\nu \cdot \frac{e^{x\left( \sqrt{1 + \frac{\nu ^2}{x^2}}\right) }}{I_\nu (x)} \\&= \Theta (1) \cdot \left( \frac{\sqrt{x^2 + \nu ^2} - \nu }{x}\right) ^\nu \cdot \frac{e^{\sqrt{x^2 + \nu ^2}}}{I_\nu (x)}, \end{aligned}$$

since Stirling’s Formula yields $(\nu /e)^\nu /\Gamma (\nu + 1/2) = \Theta (1)$.

We consider two cases, $x \le 1$ and $x > 1$.

**Case 1:** $x \le 1$. We apply Lemma 7 to Eq. (4), and find an upper bound of

$$\begin{aligned} O\left( \frac{\Gamma (\nu + 1)}{\Gamma (\nu + 1/2)}\right) = O(\sqrt{\nu }). \end{aligned}$$

**Case 2:** $x > 1$. We use Lemma 10, which yields

$$\begin{aligned} f_{\phi |R=r}(\phi )&\le \Theta (1) \cdot \sqrt{\frac{x}{\sqrt{x^2 + \nu ^2} - \nu }} \cdot \sqrt{x} = \Theta (1) \cdot \sqrt{\frac{\sqrt{x^2 + \nu ^2} + \nu }{x}}\cdot \sqrt{x} \\&= O\left( \sqrt{x} + \sqrt{\nu }\right) . \end{aligned}$$

Inserting the definitions of *x* and $\nu$ concludes the proof of the first part.

Next, let $d \ge 3$, or equivalently, $\nu \ge \frac{1}{2}$. We assume $x > 1$ in the following; the case $x \le 1$ simply follows from using Lemma 7 in Eq. (4) and dividing by $\sin \phi$.

To bound $f_{\phi |R=r}(\phi )/\sin \phi$, we follow mostly the same process. We return once more to Eq. (4), and divide by $\sin \phi$. For any fixed *x* and $\nu$, the resulting equation is then maximized when $\phi =\phi ^*$, where $\phi ^*$ satisfies

$$\begin{aligned} \sin ^2\phi ^* = \frac{2\nu -1}{x}\cos \phi ^*. \end{aligned}$$

The angle $\phi ^*$ satisfies Eqs. (6) and (7), with $\nu$ replaced by $\nu - \frac{1}{2}$. Inserting this in Eq. (4) and working through the algebra, we eventually obtain

$$\begin{aligned}&\frac{f_{\phi |R=r}(\phi )}{\sin \phi } \le \Theta (1) \cdot \frac{\left( \frac{\nu - \frac{1}{2}}{e}\right) ^{\nu -\frac{1}{2}}}{\Gamma \left( \nu + \frac{1}{2}\right) } \cdot \sqrt{x} \cdot \sqrt{\frac{\sqrt{x^2 + \left( \nu - \frac{1}{2}\right) ^2} + \nu - \frac{1}{2}}{x}}\\&\quad \cdot \left( \frac{\sqrt{x^2 + \left( \nu - \frac{1}{2}\right) ^2} - \left( \nu - \frac{1}{2}\right) }{x} \right) ^\nu \cdot \frac{\exp \left( \sqrt{x^2 + \left( \nu -\frac{1}{2}\right) ^2}\right) }{I_\nu (x)}. \end{aligned}$$

Observe that for $\nu \ge \frac{1}{2}$, we have

$$\begin{aligned} \frac{\left( \frac{\nu - \frac{1}{2}}{e}\right) ^{\nu - \frac{1}{2}}}{\Gamma \left( \nu + \frac{1}{2}\right) } \le \frac{(\nu /e)^{\nu - \frac{1}{2}}}{\Gamma \left( \nu + \frac{1}{2}\right) } = \frac{(\nu /e)^{\nu }}{\Gamma \left( \nu + \frac{1}{2}\right) } \cdot \sqrt{\frac{e}{\nu }} \in O\left( \frac{1}{\sqrt{\nu }}\right) . \end{aligned}$$

Since we assume $x > 1$, we may apply Lemma 10 to find

$$\begin{aligned}&\frac{f_{\phi |R=r}(\phi )}{\sin \phi } \le \Theta (1) \cdot \sqrt{\frac{x}{\nu }} \cdot \sqrt{\frac{\sqrt{x^2 + \left( \nu - \frac{1}{2}\right) ^2} + \nu - \frac{1}{2}}{x}} \left( \frac{\sqrt{x^2 + \left( \nu - \frac{1}{2}\right) ^2} - \left( \nu - \frac{1}{2}\right) }{x} \right) ^\nu \\&\quad \cdot \sqrt{x} \cdot \left( \frac{x}{\sqrt{x^2 + \nu ^2} - \nu } \right) ^{\nu + \frac{1}{2}}. \end{aligned}$$

Through some more elementary algebra, we can bound this (up to a constant) by

$$\begin{aligned} \frac{\sqrt{x^2 + \nu ^2} + \nu }{\sqrt{\nu }} \cdot \left( \frac{\sqrt{x^2 + \nu ^2} + \nu }{\sqrt{x^2 + \left( \nu - \frac{1}{2}\right) ^2} + \nu - \frac{1}{2}}\right) ^\nu . \end{aligned}$$

The first factor in this expression evaluates to $O(\sqrt{\nu } + x/\sqrt{\nu })$. To conclude, we must show that

$$\begin{aligned} \left( \frac{\sqrt{x^2 + \nu ^2} + \nu }{\sqrt{x^2 + \left( \nu -\frac{1}{2}\right) ^2} + \left( \nu - \frac{1}{2}\right) } \right) ^\nu \in O(1) \end{aligned}$$

for $\nu \in \{1/2, 1, 3/2, \ldots \}$. For $\nu = \frac{1}{2}$, we have

$$\begin{aligned} \left( \frac{\sqrt{x^2 + \frac{1}{4}} + \frac{1}{2}}{x}\right) ^{\frac{1}{2}} \le \sqrt{1 + \frac{1}{x}} < \sqrt{2}, \end{aligned}$$

where the latter inequality holds for $x > 1$. For $\nu \ge 1$, we use Lemma 11 to bound the given quantity by *e*. This then proves the second part of the theorem.

## Analysis of Single 2-Changes

To improve upon the previous analyses, it pays to examine where the analysis of Euclidean 2-opt with Gaussian perturbations [13] fails for $d \in \{2, 3\}$. The problem is that in the course of the proof, Manthey and Veenstra compute

$$\begin{aligned} \int _0^\infty \frac{1}{x^2}\chi _{d-1}(x)\textrm{d}{x}, \end{aligned}$$

where $\chi _d$ denotes the *d*-dimensional chi distribution. This integral is finite only when $d \ge 4$.

This problem does not appear in the results obtained by Englert et al. [8]. They consider a more general model of smoothed analysis wherein the adversary specifies a probability density for each point in the TSP instance independently. Since the only information available on the probability densities is their upper bound, they consider a simplified model of a 2-change to keep the analysis tractable. The analysis is then translated to their generic model, which incurs a factor which is super-exponential in *d*.

Even when one considers *d* to be a constant as Englert et al. do, the genericity of their model still comes at a cost when translated to a smoothed analysis with Gaussian perturbations, eventually yielding a bound which is polynomial in $\sigma ^{-d}$.

Specifying the perturbations as Gaussian enables us to analyze the true random experiment modeling a 2-change more closely, as we know the distributions of the distances between points in the smoothed instance. Combined with Theorem 5, which provides information on the angles between edges in the instance, we can carry out an analysis that improves on both Englert et al.’s as well as Manthey and Veenstra’s result when we consider Gaussian perturbations.

**Fig. 2:**
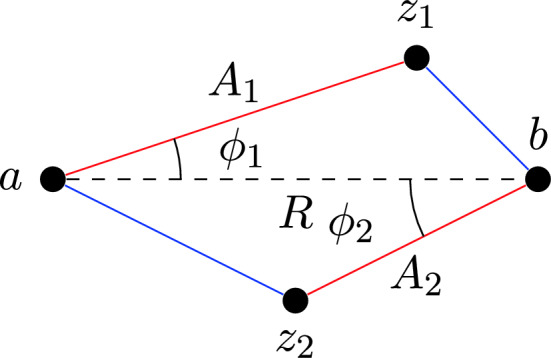
Labels of points and angles involved in a single 2-change

We first set up our model of a 2-change perturbed by Gaussian random variables. To obtain a bound for this case, we first formulate a different analysis of single 2-changes. Consider a 2-change involving the points $\{a, b, z_1, z_2\} \subseteq [-D, D]^d$, where the edges $\{a, z_1\}$ and $\{b, z_2\}$ are replaced by $\{b, z_1\}$ and $\{a, z_2\}$. The improvement to the tour length due to this 2-change is

$$\begin{aligned} \Delta = \Vert a - z_1\Vert - \Vert b - z_1\Vert + \Vert b - z_2\Vert - \Vert a - z_2\Vert . \end{aligned}$$

To analyze $\Delta$, we first define $A_1:= \Vert a - z_1\Vert$, $A_2:= \Vert b - z_2\Vert$, and $R:= \Vert a - b\Vert$. Moreover, we identify the angle $\phi _1$ as the angle between $a - z_1$ and $a - b$, and restrict it to $[0, \pi ]$. The corresponding angle $\phi _2$ is defined similarly. The restriction of these angles to $[0, \pi ]$ is without loss of generality; one may readily observe from Fig. 2 that flipping the sign of either $\phi _1$ or $\phi _2$ does not change the value of $\Delta$.

While Fig. 2 may give the impression that we are restricting the analysis to the $d = 2$ case, the analysis is valid for any $d \ge 2$. The two triangles $\triangle a z_1 b$ and $\triangle a z_2 b$ will lie in two separate planes in general. The distances involved must thus be understood as *d*-dimensional Euclidean distances.

With these definitions, we have $\Delta = \eta _1 + \eta _2$, where for $i \in [2]$

$$\begin{aligned} \eta _i = A_i - \sqrt{A_i^2 + R^2 - 2A_i R \cos \phi _i}, \end{aligned}$$

which follows from the Law of Cosines.

Suppose we condition on the events $A_1 = a_1$, $A_2 = a_2$, and $R = r$, for some $a_1, a_2, r > 0$. Under these events, $\eta _1$ and $\eta _2$ are independent random variables. Moreover, $\Delta$ is completely fixed by revealing the angles $\phi _1$ and $\phi _2$. Since we condition on $A_i = a_i$ and $R = r$, we can then bound the density of $\phi _i$ using Corollary 6.

We can use this independence to obtain bounds for $\mathbb {P}(\Delta \in (0, \epsilon ])$ for some small $\epsilon > 0$ under these events, for various orderings of $a_1$, $a_2$ and *r*. These bounds are given in Lemma 15.

We begin by obtaining a bound to the density of $\eta _i$, $i \in [2]$, using the fact that all randomness in $\eta _i$ is contained in the angle $\phi _i$ under the conditioning that $A_i = a_i$ and $R = r$. We denote by $f_{\phi _i|R=r,A_i=a_i}$ the density of the angle $\phi _i$, conditioned on $R = r$ and $A_i = a_i$.

### Lemma 12

Let $i \in [2]$. The density of $\eta _i = \Vert a - z_i\Vert - \Vert b - z_i\Vert$, conditioned on $A_i = a_i$ and $R = r$, is bounded from above by

$$\begin{aligned} \frac{a_i + r}{a_i r} \cdot \frac{f_{\phi _i|R=r,A_i=a_i}(\phi _i(\eta ))}{|\sin \phi _i(\eta )|}, \end{aligned}$$

where $$ \phi _i(\eta ) = \arccos \left( \frac{a_i^2 + r^2 - (a_i - \eta )^2}{2a_ir} \right) . $$

### Proof

Let the conditional density of $\eta _i$ be $f_{\eta _i|R=r,A_i=a_i}$. Since $\phi _i$ is restricted to $[0, \pi ]$ by assumption, there exists a bijection between $\eta _i$ and $\phi _i$. To be precise, we have

$$\begin{aligned} \phi _i(\eta _i) = \arccos \left( \frac{a_i^2 + r^2 - (a_i - \eta _i)^2}{2a_ir} \right) . \end{aligned}$$

By standard transformation rules of probability densities, it holds that

$$\begin{aligned} f_{\eta _i|R=r,A=a_i}(\eta ) = \left| \frac{\textrm{d}\phi _i(\eta )}{\textrm{d}\eta }\right| f_{\phi _i|R=r,A_i=a_i}(\phi _i(\eta )). \end{aligned}$$

The derivative is easily evaluated:

$$\begin{aligned} \frac{\textrm{d}\phi _i(\eta )}{\textrm{d}\eta } = \frac{-1}{\sqrt{1 - \left( \frac{a_i^2 + r^2 - (a_i-\eta )^2}{2a_i r}\right) }} \cdot \frac{a_i - \eta }{a_i r} = \frac{-1}{\sin \phi (\eta )} \cdot \frac{a_i-\eta }{a_i r}. \end{aligned}$$

Finally, we have $a_i - \eta \le a_i + r$, which follows from the triangle inequality. This concludes the proof. $\square$

With Corollary 6, we have an upper bound for $f_{\phi _i|R=r,A_i = a_i}$. Unfortunately, simply inserting this upper bound is not enough for us to bound $f_{\eta _i|A_i=a_i,R=r}$, since the density as obtained from Lemma 12 diverges for $\phi = 0$ and $\phi = \pi$. There is however a way to cure this divergence.

We now consider a full 2-change (cf. Fig. 2). To analyze the improvement $\Delta$ caused by this 2-change, we construct a random experiment, conditioned on the outcomes $A_1 = a_1$, $A_2 = a_2$, and $R = r$. We write this random experiment in Algorithm 1, since we will need to execute different experiments depending on the ordering of the values of $a_1$, $a_2$ and *r*. The parameters $b_1$ and $b_2$ of this algorithm will take values in $\{a_1, a_2, r\}$, depending on this ordering.

**Algorithm 1:**
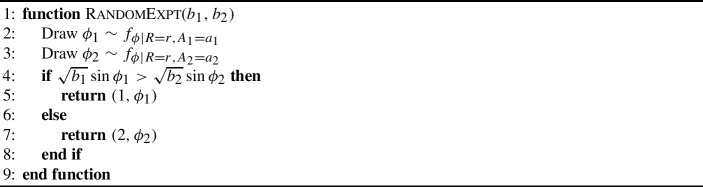
The algorithm we use to model a random 2-change with fixed $A_1 = a_1$, $A_2 = a_2$, and $R = r$.

The function RandomExpt outlined in Algorithm 1 branches on the outcome of the variable $Z_i = \sqrt{b_i}\sin \phi _i$, $i \in [2]$, where $b_i$ is some distance; we will choose $b_i$ among $\{r, a_i\}$ in subsequent lemmas.

Note that RandomExpt returns a tuple $(i, \phi )$, where $i \in [2]$. We call the angle returned by RandomExpt the *good angle*. Moreover, we label the event $i = 1$ as $E_1$, and $i = 2$ by $E_2$. The crux of the analysis is now to analyze $\eta _1$ if $E_1$ occurs, and $\eta _2$ if $E_2$ occurs, as under $E_i$ the density of $\eta _i$ is bounded from above.

### Lemma 13

Let $(i, \phi ) = \texttt {RandomExpt}(b_1, b_2)$ for some $b_1, b_2 > 0$. Let $j = 3 - i$. The density of $\phi$, conditioned on $R = r$, $A_1 = a_1$, $A_2 = a_2$, is then bounded from above by

$$\begin{aligned} \frac{2M_{\phi _1}M_{\phi _2}}{\mathbb {P}(E_i)} \cdot \arcsin \left( \min \left\{ 1, \sqrt{\frac{b_i}{b_j}}\sin \phi \right\} \right) , \end{aligned}$$

where $$M_{\phi _i} = \max _{0 \le \phi \le \pi } f_{\phi _i|R=r,A_i=a_i}(\phi )$$.

### Proof

We omit the conditioning on $A_1 = a_1$, $A_2 = a_2$ and $R = r$ in the following, for the sake of clarity. We prove only the case $i = 1$, thus conditioning on $E_1$, as the proof for $i = 2$ proceeds essentially identically.

Let $X_i = \sqrt{b_i}\sin \phi _i$, $i \in [2]$. The event $E_1$ is then equivalent to $X_1 > X_2$. Let *Z* in turn denote the random variable given by $X_1$ conditioned on $E_1$. The cumulative distribution function of *Z* is equal to

$$\begin{aligned} F_Z(x) = \mathbb {P}(X_1 \le x |X_1> X_2) = \frac{\mathbb {P}(X_1 \le x \wedge X_1 > X_2)}{\mathbb {P}(E_1)}. \end{aligned}$$

By the independence of $X_1$ and $X_2$, this is equal to

$$\begin{aligned} F_Z(x) = \frac{1}{\mathbb {P}(E_1)} \cdot \int _0^x f_{X_1}(y) \int _0^y f_{X_2}(z)\textrm{d}z \textrm{d}y. \end{aligned}$$

Computing the density of *Z* is then simply a matter of differentiation. Since $\mathbb {P}(E_1)$ does not depend on *x*, we obtain

$$\begin{aligned} f_Z(x) = \frac{1}{\mathbb {P}(E_1)} \cdot f_{X_1}(x)\int _0^x f_{X_2}(z)\textrm{d}z. \end{aligned}$$

We next require the density of $X_i = \sqrt{b_i}\sin \phi _i$. Observe that

8 $$\begin{aligned} \mathbb {P}(X_i \le x) = \mathbb {P}\left( \phi _i \le \arcsin (x/\sqrt{b_i})\right) + \mathbb {P}\left( \phi _i \ge \pi - \arcsin (x/\sqrt{b_i})\right) . \end{aligned}$$

Differentiating this expression to *x*, we find for $x < \sqrt{b_i}$

$$\begin{aligned} f_{X_i}(x)&= \frac{\textrm{d}}{\textrm{d}x} \left( \mathbb {P}\left( \phi _i \le \arcsin (x/\sqrt{b_i})\right) + 1 - \mathbb {P}\left( \phi _i \ge \pi - \arcsin (x/\sqrt{b_i})\right) \right) \\&= \frac{\textrm{d}}{\textrm{d}x}\left( \arcsin \left( \frac{x}{\sqrt{b_i}}\right) \right) \cdot \left[ f_{\phi _i}\left( \arcsin \left( \frac{x}{\sqrt{b_i}}\right) \right) + f_{\phi _i}\left( \pi - \arcsin \left( \frac{x}{\sqrt{b_i}}\right) \right) \right] \\&= \frac{1}{\sqrt{b_i - x^2}} \cdot \left[ f_{\phi _i}\left( \arcsin \left( \frac{x}{\sqrt{b_i}}\right) \right) + f_{\phi _i}\left( \pi - \arcsin \left( \frac{x}{\sqrt{b_i}}\right) \right) \right] , \end{aligned}$$

and 0 for $x \ge \sqrt{b_i}$. Letting $M_{\phi _i} = \max _{0 \le \phi \le \pi } f_{\phi _i|R=r,A_i=a_i}(\phi )$, which exists by Corollary 6, we obtain

$$\begin{aligned} f_{X_i}(x) \le 2M_{\phi _i}\cdot {\left\{ \begin{array}{ll} \frac{1}{\sqrt{b_i - x^2}}, &  \text {if } x < \sqrt{b_i}, \\ 0, &  \text {otherwise.} \end{array}\right. } \end{aligned}$$

Using this density, together with the identity $\int _0^x (\sqrt{b} - y^2)^{-1/2}\textrm{d}y = \arcsin (x/\sqrt{b})$ for $x < \sqrt{b}$, we obtain

$$\begin{aligned} f_{Z}(x) \le \frac{2M_{\phi _1}M_{\phi _2}}{\mathbb {P}(E_1)} \cdot \frac{\arcsin \left( \min \left\{ 1, \frac{x}{\sqrt{b_2}}\right\} \right) }{\sqrt{b_1 - x^2}} \end{aligned}$$

if $x < \sqrt{b_1}$, and $f_Z(x) = 0$ otherwise. It remains to convert *Z* back to $\phi$, where $\phi$ is the good angle. Since we have conditioned on $E_1$, we know that $Z = \sqrt{b_1}\sin \phi$. Using similar considerations as used in Eq. (8), we have

$$\begin{aligned} f_Z(x) = \frac{1}{\sqrt{b_1 - x^2}}f_{\phi }(\arcsin (x/\sqrt{b_1})) +\frac{1}{\sqrt{b_1 - x^2}}f_{\phi }(\pi - \arcsin (x/\sqrt{b_1})). \end{aligned}$$

Since this expression holds for all $x \in (0, \sqrt{b_1})$, and since probability densities are non-negative, it follows that

$$\begin{aligned} f_{\phi }(\phi ) \le \frac{2M_{\phi _1}M_{\phi _2}}{\mathbb {P}(E_1)} \cdot \arcsin \left( \min \left\{ 1, \sqrt{\frac{b_1}{b_2}}\sin \phi \right\} \right) , \end{aligned}$$

for all $$\phi \in (0, \pi )$$. $$\square $$

For the next part, we apply Lemmas 13–12 to bound the density of $\eta _i$, given that $E_i$ occurs.

### Lemma 14

Let $i \in [2]$ and $j = 3 - i$. Let $f_{\eta _i|E_i}$ denote the density of $\eta _i$, conditioned on $E_i$ as well as the outcomes $R = r$, $A_1 = a_1$, and $A_2 = a_2$. Then

$$\begin{aligned} f_{\eta _i|E_i}(\eta ) \le \frac{1}{\mathbb {P}(E_i)} \cdot \frac{2\pi M_{\phi _1} M_{\phi _2}}{\min \{a_1, r\}\min \{a_2, r\}}, \end{aligned}$$

where $$M_{\phi _i} = \max _{0 \le \phi \le \pi } f_{\phi _i|R=r,A_i=a_i}(\phi )$$.

### Proof

We prove only the case $i = 1$. From Lemma 12, we know that

$$\begin{aligned} f_{\eta _i|E_i}(\eta ) \le \frac{a_i + r}{a_i r} \cdot \frac{f_{\phi _i|E_i,A_1=a_1,A_2=a_2}(\phi )}{\sin \phi }. \end{aligned}$$

Let $(i, \phi ) = \texttt {RandomExpt}(b_1, b_2)$, for some $b_1, b_2 > 0$. We will choose values for $b_1$ and $b_2$ depending on the ordering of $a_1, a_2$ and *r*. Note that we may do this, since we know the choices of $a_1$, $a_2$ and *r* before executing RandomExpt.

Since we condition on $E_1$, we know that $i = 1$, and hence that $\phi _1$ is the good angle. By Lemma 13, we can obtain a bound for $f_{\phi |E_i,A_1=a_1,A_2=a_2,R=r}$. We thus find

$$\begin{aligned} f_{\eta _1|E_1}(\eta ) \le \frac{2M_{\phi _1}M_{\phi _2}}{\mathbb {P}(E_1)} \cdot \frac{a_1 + r}{a_1 r} \cdot \frac{\arcsin \left( \min \left\{ 1, \sqrt{\frac{b_1}{b_2}}\sin \phi \right\} \right) }{\sin \phi }. \end{aligned}$$

First, suppose $\sin \phi \ge \sqrt{b_2/b_1}$. Then the arcsine evaluates to $\pi /2$, and so the above is bounded from above by

$$\begin{aligned} \frac{\pi }{2}\sqrt{\frac{b_1}{b_2}}. \end{aligned}$$

Second, suppose $\sin \phi < \sqrt{b_2/b_1}$. Since $\arcsin (x) \le \pi x/2$ for $x \in (0, 1)$, this case yields the same bound, and we obtain

$$\begin{aligned} f_{\eta _1|E_1}(\eta ) \le \frac{\pi M_{\phi _1}M_{\phi _2}}{\mathbb {P}(E_1)} \cdot \frac{a_1 + r}{a_1 r} \cdot \sqrt{\frac{b_1}{b_2}} \end{aligned}$$

We now examine the four relevant orderings of $a_1$, $a_2$ and *r*.

**Case 1:** $a_1, a_2 \le r$. We let $b_1 = a_1$ and $b_2 = a_2$. Then we have

$$\begin{aligned} \frac{a_1 + r}{a_1 r} \cdot \sqrt{\frac{a_1}{a_2}} = \frac{a_1 + r}{r\sqrt{a_1 a_2}} \le \frac{2r}{r\sqrt{a_1 a_2}} = \frac{2}{\sqrt{a_1 a_2}}. \end{aligned}$$

**Case 2:** $a_1, a_2 \ge r$. We let $b_1 = b_2 = r$, and obtain

$$\begin{aligned} \frac{a_1 + r}{a_1 r} \le \frac{2a_1}{a_1 r} = \frac{2}{r}. \end{aligned}$$

**Case 3:** $a_1 \ge r \ge a_2$. We let $b_1 = r$ and $b_2 = a_2$, which yields

$$\begin{aligned} \frac{a_1 + r}{a_1 r} \cdot \sqrt{\frac{r}{a_2}} = \frac{a_1 + r}{\sqrt{a_2 r} a_1} \le \frac{2}{\sqrt{a_2 r}}. \end{aligned}$$

**Case 4:** $a_2 \ge r \ge a_1$. We let $b_1 = a_1$ and $b_2 = r$, to find

$$\begin{aligned} \frac{a_1 + r}{a_1 r} \sqrt{\frac{a_1}{r}} \le \frac{2r\sqrt{a_1}}{a_1 r\sqrt{r}} = \frac{2}{\sqrt{a_1 r}}. \end{aligned}$$

This final case concludes the proof. $\square$

The bound on the density of $\eta _i$ from Lemma 14 puts us in the position to prove a bound on the probability that $\Delta \in (0,\epsilon ]$.

### Lemma 15

Let $\Delta$ denote the improvement of a 2-change. Then

$$\begin{aligned} \mathbb {P}(\Delta \in (0, \epsilon ] |A_1 = a_1, A_2 = a_2, R = r) \le \frac{\pi M_{\phi _1} M_{\phi _2}\epsilon }{\min \{a_1, r\}\min \{a_2, r\}}, \end{aligned}$$

where $$M_{\phi _i} = \max _{0 \le \phi \le \pi } f_{\phi _i|R=r,A_i=a_i}(\phi )$$.

### Proof

We condition first on $E_1$, and then let an adversary choose an outcome for $\eta _2$, say, $\eta _2 = t$. Then we have $\Delta \in (0, \epsilon ]$ iff $\eta _1 \in (-t, -t+\epsilon ]$, which is an interval of size $\epsilon$.

Since the probability that $\eta _1$ falls into an interval of size $\epsilon$ is at most $\epsilon \cdot \max _{\eta } f_{\eta _1|E_1}(\eta )$, all we need to conclude the proof for $E_1$ is a bound on $f_{\eta _1|E_1}(\eta )$. This is provided by Lemma 14.

We then repeat the same argument for $E_2$. The result is obtained by applying the Law of Total Probability. $\square$

With Lemma 15, we could prove a bound on the smoothed complexity of 2-opt already. However, the resulting bound would be weaker than existing results. Instead of analyzing single 2-changes, we thus use the framework of linked pairs of 2-changes in Sect. 4.

For the analysis in Sect. 4, it is convenient to have some lemmas similar to Lemma 15, with one or more of the distances $A_1$, $A_2$ and *R* integrated out. These are given in Lemmas 16–18. The proofs are straightforward computations.

### Lemma 16

For $$i \in [2]$$,

$$\begin{aligned}  &   \mathbb {P}(\Delta \in (0, \epsilon ] |A_i = a_i, R = r)\\  &   = O\left( \left( \frac{\sqrt{d}D}{\sigma ^2} + \frac{d}{\sqrt{a_i r}} + \frac{d}{r} + \frac{d^{3/4}\sqrt{D}}{\sigma } \left( \frac{1}{\sqrt{a_i}} + \frac{1}{\sqrt{r}} \right) \right) \cdot \epsilon \right) . \end{aligned}$$

### Proof

We assume $i = 1$, since by symmetry the result for $i = 2$ follows essentially identically.

Consider the cases $a_1 \le r$ and $a_1 \ge r$ separately.

**Case 1:** $a_1 \le r$. For this case, we have by Lemma 15 for some constants $c, c', c'' > 0$,

$$\begin{aligned} \mathbb {P}(\Delta \in (0, \epsilon ]|A_1 = a_1, A_2=a_2,R=r)&\le c\cdot \frac{M_{\phi _1}M_{\phi _2}\epsilon }{\sqrt{a_1}} \cdot {\left\{ \begin{array}{ll} \frac{1}{\sqrt{r}}, &  \text {if } a_2 \ge r \\ \frac{1}{\sqrt{a_2}}, &  \text {if } a_2 \le r \end{array}\right. }\\&\le c' \cdot \frac{M_{\phi _1}\epsilon }{\sqrt{a_1}} \cdot {\left\{ \begin{array}{ll} \sqrt{\frac{d}{r}} + \frac{d^{1/4}\sqrt{D}}{\sigma }, &  \text {if } a_2 \ge r \\ \sqrt{\frac{d}{a_2}} + \frac{d^{1/4}\sqrt{D}}{\sigma }, &  \text {if } a_2 \le r \end{array}\right. } \\&\le c'' \cdot \frac{M_{\phi _1}\epsilon }{\sqrt{a_1}} \left( \sqrt{\frac{d}{r}} + \sqrt{\frac{d}{a_2}} + \frac{d^{1/4}\sqrt{D}}{\sigma }\right) , \end{aligned}$$

where we use Corollary 6 to bound $M_{\phi _2}$.

We can now use Lemmas 4 and 3 to integrate out $a_2$, leaving us with

$$\begin{aligned} O\left( \frac{M_{\phi _1}\epsilon }{\sqrt{a_1}} \left( \sqrt{\frac{d}{r}} + \frac{d^{1/4}}{\sqrt{\sigma }} + \frac{d^{1/4}\sqrt{D}}{\sigma } \right) \right) . \end{aligned}$$

Using that $D \ge 1$ and $\sigma \le 1$, we see that the third term in the inner brackets is at least as large as the second term, and so we obtain

$$\begin{aligned} \mathbb {P}(\Delta \in (0, \epsilon ]|A_1 = a_1, A_2=a_2,R=r) = O\left( \frac{M_{\phi _1}}{\sqrt{a_1}}\left( \sqrt{\frac{d}{r}} + \frac{d^{1/4}\sqrt{D}}{\sigma }\right) \cdot \epsilon \right) . \end{aligned}$$

Now we use Corollary 6 to conclude $M_{\phi _1} = O\left( d^{1/4}\sqrt{Da_1}/\sigma \right)$, yielding

$$\begin{aligned}&O\left( \left( \sqrt{\frac{d}{a_1}} + \frac{d^{1/4}\sqrt{D}}{\sigma }\right) \cdot \left( \sqrt{\frac{d}{r}} + \frac{d^{1/4}\sqrt{D}}{\sigma }\right) \cdot \epsilon \right) \\&= O\left( \left( \frac{d}{\sqrt{a_1 r}} + \frac{d^{3/4}\sqrt{D}}{\sigma }\left( \frac{1}{\sqrt{a_1}} + \frac{1}{\sqrt{r}} \right) + \frac{\sqrt{d}D}{\sigma ^2} \right) \cdot \epsilon \right) . \end{aligned}$$

**Case 2:** $a_1 \ge r$. Here, Lemma 15 tells us

$$\begin{aligned} \mathbb {P}(\Delta \in (0, \epsilon ]|A_1 = a_1, A_2=a_2,R_1=r))&\le c\cdot M_{\phi _1}M_{\phi _2} \epsilon \cdot {\left\{ \begin{array}{ll} \frac{1}{r}, &  \text {if } a_2 \ge r \\ \frac{1}{\sqrt{r a_2}}, &  \text {if } a_2 \le r \end{array}\right. } \\&\le c' \cdot M_{\phi _1}\epsilon \cdot {\left\{ \begin{array}{ll} \frac{\sqrt{d}}{r} + \frac{d^{1/4}\sqrt{D}}{\sigma \sqrt{r}}, &  \text {if } a_2 \ge r, \\ \sqrt{\frac{d}{r a_2}} + \frac{d^{1/4}\sqrt{D}}{\sigma \sqrt{r}}, &  \text {if } a_2 \le r \end{array}\right. } \\&\le c'' \cdot M_{\phi _1} \epsilon \cdot \left( \frac{\sqrt{d}}{r} + \sqrt{\frac{d}{ra_2}} + \frac{d^{1/4}\sqrt{D}}{\sigma \sqrt{r}} \right) , \end{aligned}$$

again for some $c, c', c'' > 0$ and using Corollary 6 to bound $M_{\phi _2}$.

Integrating out $a_2$ using Lemmas 4 and 3, we have

$$\begin{aligned} O\left( M_{\phi _1}\epsilon \cdot \left( \frac{\sqrt{d}}{r} + \frac{d^{1/4}}{\sqrt{\sigma r}} + \frac{d^{1/4}\sqrt{D}}{\sigma \sqrt{r}} \right) \right) \subseteq O\left( M_{\phi _1}\epsilon \cdot \left( \frac{\sqrt{d}}{r} + \frac{d^{1/4}\sqrt{D}}{\sigma \sqrt{r}} \right) \right) . \end{aligned}$$

Using Corollary 6 to insert $M_{\phi _1} = O\left( \sqrt{d} + d^{1/4}\sqrt{Dr}/\sigma \right)$, we find

$$\begin{aligned}  &   O\left( \left( \frac{\sqrt{d}}{r} + \frac{d^{1/4}\sqrt{D}}{\sigma \sqrt{r}} \right) \cdot \left( \sqrt{d} + \frac{d^{1/4}\sqrt{Dr}}{\sigma } \right) \cdot \epsilon \right) \\  &   = O\left( \left( \frac{d}{r} + \frac{d^{3/4}\sqrt{D}}{\sigma \sqrt{r}} + \frac{\sqrt{d}D}{\sigma ^2} \right) \cdot \epsilon \right) . \end{aligned}$$

The result follows from these two cases. $\square$

### Lemma 17

For $$i \in [2]$$,

$$\begin{aligned} \mathbb {P}(\Delta \in (0,\epsilon ] |A_i = a_i) = O\left( \left( \frac{\sqrt{d}D}{\sigma ^2} + \frac{d^{3/4}\sqrt{D}}{\sigma \sqrt{a_i}} \right) \cdot \epsilon \right) . \end{aligned}$$

### Proof

From Lemma 16, we have

$$\begin{aligned}  &   \mathbb {P}(\Delta \in (0, \epsilon ] |A_i = a_i, R = r)\\  &   = O\left( \left( \frac{\sqrt{d}D}{\sigma ^2} + \frac{d}{\sqrt{a_i r}} + \frac{d}{r} + \frac{d^{3/4}\sqrt{D}}{\sigma } \left( \frac{1}{\sqrt{a_i}} + \frac{1}{\sqrt{r}} \right) \right) \cdot \epsilon \right) . \end{aligned}$$

We can then apply Lemmas 4 and 3 to integrate out *r*. This leaves

$$\begin{aligned}  &   O\left( \left( \frac{\sqrt{d}D}{\sigma ^2} + \frac{d^{1/4}}{\sqrt{a_i\sigma }} + \frac{\sqrt{d}}{\sigma } + \frac{\sqrt{dD}}{\sigma ^{3/2}} + \frac{d^{3/4}\sqrt{D}}{\sigma \sqrt{a_i}} \right) \cdot \epsilon \right) \\  &   \subseteq O\left( \left( \frac{\sqrt{d}D}{\sigma ^2} + \frac{d^{3/4}\sqrt{D}}{\sigma \sqrt{a_i}} \right) \cdot \epsilon \right) , \end{aligned}$$

as claimed. $$\square $$

### Lemma 18

$$\begin{aligned} \mathbb {P}(\Delta \in (0, \epsilon ] |R = r) = O\left( \left( \frac{\sqrt{d}D}{\sigma ^2} + \frac{d}{r} + \frac{d^{3/4}\sqrt{D}}{\sigma \sqrt{r}} \right) \cdot \epsilon \right) . \end{aligned}$$

### Proof

The result follows from taking Lemma 17 and integrating out $a_i$ using Lemmas 4 and 3. $\square$

## Linked Pairs of 2-Changes

To obtain bounds on the smoothed complexity of 2-opt, we consider so-called linked pairs of 2-changes, introduced previously by Englert et al. [8]. A pair of 2-changes is said to be linked if some edge removed from the tour by one 2-change is added to the tour by the other 2-change.

Such linked pairs have been considered in several previous works [8, 13]. In each case, the distinction has been made between several types of linked pairs. In our analysis, only two of these types are relevant, and so we will describe only these types for the sake of brevity.

We consider 2-changes which share exactly one edge, and subdivide them into pairs of type 0 and of type 1. A generic 2-change removes the edges $\{z_1, z_2\}$ and $\{z_3, z_6\}$ while adding $\{z_1, z_6\}$ and $\{z_2, z_3\}$. The other 2-change removes $\{z_3, z_4\}$ and $\{z_5, z_6\}$ while adding $\{z_3, z_6\}$ and $\{z_4, z_5\}$. Note that $\{z_3, z_6\}$ occurs in both 2-changes.

- If $|\{z_1, \ldots , z_6\}| = 6$, then we say the linked pair is of type 0.
- If $|\{z_1, \ldots , z_6\}| = 5$, then we say the linked pair is of type 1.

Type 1 can itself be subdivided into two types, 1a and 1b. We will detail this distinction in Sect. 4.2.

Before moving on to analyzing linked pairs, we state a useful lemma that justifies limiting the discussion to just linked pairs of types 0 and 1.

### Lemma 19

[8, Lemma 9] In every sequence of *t* consecutive 2-changes the number of disjoint pairs of 2-changes of type 0 or type 1 is at least $\Omega (t) - O(n^2)$.

### Type 0

**Fig. 3:**
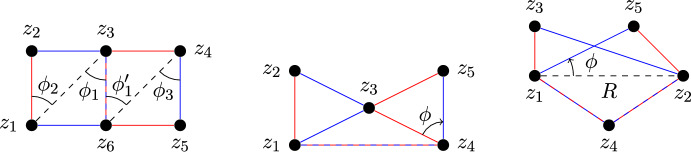
Labels of points involved in the three types of pairs of linked 2-changes. Left: type 0. Center: type 1a. Right: type 1b

We begin with type 0, as this is by far the simplest linked pair. For clarity, see Fig. 3 (left) for an illustration of a type 0 linked pair. It should be noted that, while Fig. 3 shows a specific configuration of vertices in two dimensions, the results of this section hold generally; the analysis does not depend on any point having a particular orientation with respect to its neighbors. The same holds for the results in Sect. 4.2.

The improvement of a type 0 linked pair is completely specified by a small number of random variables. We require five distances between vertices, $R_1 = \Vert z_1 - z_3\Vert$, $A_1 = \Vert z_3 - z_6\Vert$, $A_2 = \Vert z_1 - z_2\Vert$, $R_2 = \Vert z_4 - z_6\Vert$ $A_3 = \Vert z_4 - z_5\Vert$. Additionally, we need the following angles:

1. $$\phi _1$$ between $$z_2-z_1$$ and $$z_3-z_1$$,
2. $$\phi _2$$ between $$z_1-z_3$$ and $$z_6-z_3$$,
3. $$\phi _1'$$ between $$z_3-z_6$$ and $$z_4-z_6$$,
4. $$\phi _3$$ between $$z_6-z_4$$ and $$z_5-z_4$$.

Note that, if we condition on $A_1 = a_1$, the events $\Delta _1 \in (0, \epsilon ]$ and $\Delta _2 \in (0, \epsilon ]$ are independent. We can then apply Lemma 15, together with several applications of Lemma 4.

#### Lemma 20

Let $\Delta ^{\textrm{link}}_\textrm{min}$ denote the minimum improvement of any type 0 pair of linked 2-changes, and assume that $\mathcal {X}\subseteq [-D, D]^d$. Then

$$\begin{aligned} \mathbb {P}(\Delta ^\textrm{link}_{\textrm{min}} \in (0, \epsilon ]) = O\left( \frac{d D^2 n^6 \epsilon ^2}{\sigma ^4} \right) . \end{aligned}$$

#### Proof

The result follows from the independence of $\Delta _1$ and $\Delta _2$ when conditioning on $A_1 = a_1$. Observe that $\mathbb {P}(\Delta ^\textrm{link} \in (0, \epsilon ]) \le \mathbb {P}(\Delta _1 \in (0, \epsilon ] \wedge \Delta _2 \in (0,\epsilon ])$. Thus, using Lemma 17,

$$\begin{aligned} \mathbb {P}(\Delta ^\textrm{link} \in (0,\epsilon ] |A_1 = a_1) = O\left( \left( \frac{\sqrt{d}D}{\sigma ^2} + \frac{d^{3/4}\sqrt{D}}{\sigma \sqrt{a_1}} \right) ^2 \epsilon ^2 \right) . \end{aligned}$$

Straightforward algebra yields

$$\begin{aligned} \left( \frac{\sqrt{d}D}{\sigma ^2} + \frac{d^{3/4}\sqrt{D}}{\sigma \sqrt{a_1}} \right) ^2 = O\left( \frac{dD^2}{\sigma ^4} + \frac{d^{3/2}D}{\sigma ^2 a_1} + \frac{d^{5/4}D^{3/2}}{\sigma ^3\sqrt{a_1}} \right) . \end{aligned}$$

Using Lemmas 3 and 4 to integrate out $a_1$, we obtain

$$\begin{aligned} \frac{dD^2}{\sigma ^4} + \frac{dD}{\sigma ^3} + \frac{dD^{3/2}}{\sigma ^{7/2}} = O\left( \frac{dD^2}{\sigma ^4} \right) . \end{aligned}$$

Taking a union bound over the $O(n^6)$ different type 0 pairs completes the proof. $\square$

### Type 1

As mentioned previously, type 1 linked pairs can be subdivided into two distinct subtypes. Subtype 1a shares exactly one edge between the two 2-changes, while subtype 1b shares two edges.

#### Type 1a

We first consider type 1a. See Fig. 3 (center) for a graphical representation of the type, as well as the labels of the points and edges involved.

Let the 2-change replacing $\{z_1, z_2\}$ and $\{z_3, z_4\}$ by $\{z_2, z_3\}$ and $\{z_1, z_4\}$ be called $S_1$, and the 2-change replacing $\{z_1, z_4\}$ and $\{z_3, z_5\}$ by $\{z_1, z_3\}$ and $\{z_4, z_5\}$ be called $S_2$.

We proceed by conditioning on $A_2 = \Vert z_3 - z_4\Vert = a_2$ and $A_3 = \Vert z_4 - z_5\Vert = a_3$. Using Lemma 15, we can then compute the probability that $\Delta _1 \in (0, \epsilon ]$. Moreover, the location of $z_5$ is then still random. Hence, the random variable $\eta = \Vert z_3 - z_5\Vert - \Vert z_4 - z_5\Vert$ can be analyzed independently from $\Delta _1$.

For the density of $\eta$, we have the following lemma from Englert et al. [8].

##### Lemma 21

[8, Lemma 15, modified] Let $i \in [2]$, and assume that $\mathcal {X}\subseteq [-D, D]^d$. For $a_2, a_3 \in (0, 2\sqrt{d}D]$ and $\eta \in (-a_2, \min \{a_2, 2a_3 - a_2\})$,

$$\begin{aligned} f_{\eta |A_2 = a_2, A_3=a_3}(\eta ) \le M_{\phi } \cdot {\left\{ \begin{array}{ll} \sqrt{\frac{2}{a_2^2 - \eta ^2}}, &  \text {if } a_3 \ge a_2, \\ \sqrt{\frac{2}{(a_2 + \eta )(2a_3 - a_2 - \eta }}, &  \text {if } a_3 < a_2, \end{array}\right. } \end{aligned}$$

where $M_\phi = \max _{0 \le \phi \le \pi } f_{\phi |A_2=a_2,A_3=a_3}(\phi )$. For $\eta \notin (-r, \min \{a_2, 2a_3 - a_2\})$, the density vanishes.

Note that the factor $M_{\phi }$ was not present in the original statement of Lemma 21. This is because the original statement concerned a simplified random experiment, wherein the points $z_5$ and $z_3$ are chosen uniformly from a hyperball centered on $z_4$. As such, $\phi$ is assumed to be distributed uniformly.^1^ Since we do not analyze a simplified random experiment, we cannot make this assumption. However, examining the original proof of Lemma 21, this can be resolved by simply inserting the upper bound of the density of $\phi$, conditioned on $A_2 = a_2$ and $A_3 = a_3$. This bound is provided to us by Corollary 6.

##### Lemma 22

Let $\Delta _2$ be the improvement yielded by $S_2$, and assume that $\mathcal {X}\subseteq [-D, D]^d$. Then

$$\begin{aligned} \mathbb {P}(\Delta _2 \in (0, \epsilon ] |A_2 = a_2) = O\left( \left( \frac{d^{1/4}\sqrt{D}}{\sigma } + \sqrt{\frac{d}{a_2}} \right) \cdot \sqrt{\epsilon } \right) . \end{aligned}$$

##### Proof

We obtain the density of $\eta$ from Lemma 21. As before, we need to subdivide into the cases $a_2 \le a_3$ and $a_2 \ge a_3$.

**Case 1:** $a_3 \le a_2$. For this case, the conditional density of $\eta$ reads

$$\begin{aligned} f_{\eta |A_2=a_2, A_3=a_3}(\eta ) \le M_{\phi } \cdot {\left\{ \begin{array}{ll} \sqrt{\frac{2}{a_3(a_2 + \eta )}}, &  \eta \le a_3 - a_2, \\ \sqrt{\frac{2}{a_3(2a_3 - a_2 - \eta }}, &  \eta \ge a_3 - a_2. \end{array}\right. } \end{aligned}$$

We assume that the random variable $\Vert z_1 - z_4\Vert - \Vert z_1 - z_3\Vert$ has been fixed by the adversary. This fixes an interval of size $\epsilon$ for $\eta$ to fall within, should $\Delta _2 \in (0,\epsilon ]$ occur. Observe that $f_{\eta |A_2=a_2,A_3=a_3}$ integrated over any interval of size $\epsilon$ yields at most $O(M_\phi \sqrt{\epsilon /a_3})$. Since $a_3 \le a_2$, we have $M_{\phi } = O(\sqrt{d} + d^{1/4}\sqrt{Da_3}/\sigma )$. Thus, for any interval *I* of size $\epsilon$,

$$\begin{aligned} \mathbb {P}(\eta \in I |A_2 = a_2, A_3 = a_3) = O\left( \left( \sqrt{\frac{d}{a_3}} + \frac{d^{1/4}\sqrt{D}}{\sigma }\right) \cdot \sqrt{\epsilon } \right) . \end{aligned}$$

**Case 2:** $a_3 \ge a_2$. For this case, we have

$$\begin{aligned} f_{\eta |A_2 = a_2, A_3 = a_3}(\eta ) = M_\phi \sqrt{\frac{2}{a_2}} \cdot \sqrt{\frac{1}{a_2 - |\eta |}}. \end{aligned}$$

Similarly as in Case 1, this function integrates to at most $O(M_\phi \sqrt{\epsilon /a_2})$. Here, we have $M_{\phi } = O(\sqrt{d} + d^{1/4}\sqrt{Da_2}/\sigma )$, so we obtain

$$\begin{aligned} \mathbb {P}(\eta \in I |A_2 = a_2, A_3 = a_3) = O\left( \left( \sqrt{\frac{d}{a_2}} + \frac{d^{1/4}\sqrt{D}}{\sigma }\right) \cdot \sqrt{\epsilon } \right) . \end{aligned}$$

Combining the two cases above, we see that

$$\begin{aligned} \mathbb {P}(\Delta _2 \in (0,\epsilon ]|A_2 = a_2, A_3 = a_3) = O\left( \left( \frac{d^{1/4}\sqrt{D}}{\sigma } + \sqrt{\frac{d}{a_2}} + \sqrt{\frac{d}{a_3}} \right) \cdot \sqrt{\epsilon } \right) . \end{aligned}$$

We can now integrate out $a_3$ using Lemmas 4 and 3. Then, using $D \ge 1$, $d \ge 2$ and $\sigma \le 1$, we eventually arrive at the stated result. $\square$

Using Lemmas 4 and 22, we can easily prove the following statement about type 1a pairs of 2-changes.

##### Lemma 23

Let $\Delta ^{\textrm{link}}_\textrm{min}$ denote the minimum improvement of any type 1a pair of 2-changes, and assume that $\mathcal {X}\subseteq [-D, D]^d$. Then

$$\begin{aligned} \mathbb {P}(\Delta ^{\textrm{link}}_\textrm{min} \in (0,\epsilon ]) = O\left( \frac{n^5 d^{3/4}D^{3/2}}{\sigma ^{3}} \epsilon ^{3/2} \right) . \end{aligned}$$

##### Proof

As in the proof of Lemma 20, we can simply use Lemmas 17 and 22 to compute the probability that both $\Delta _1 \in (0, \epsilon ]$ and $\Delta _2 \in (0, \epsilon ]$, which bounds the probability that $\Delta _1 + \Delta _2 \in (0,\epsilon ]$:

$$\begin{aligned} \mathbb {P}(\Delta _1, \Delta _2 \in (0, \epsilon ] |A_2 = a_2)&= O\left( \left( \frac{d^{3/4}D^{3/2}}{\sigma ^3} + \frac{dD}{\sigma ^2\sqrt{a_2}} + \frac{d^{5/4}\sqrt{D}}{\sigma a_2} \right) \cdot \epsilon ^{3/2} \right) . \end{aligned}$$

Using Lemmas 4 and 3, with $d \ge 2$, $D \ge 1$ and $\sigma \le 1$ in conjunction with a union bound over the $O(n^5)$ pairs of type 1a yields the result. $\square$

#### Type 1b

The final type of linked pair we consider is type 1b. See Fig. 3 (right) for a graphical representation.

Let $S_1$ denote the 2-change replacing $\{z_1, z_3\}$ and $\{z_2, z_4\}$ with $\{z_2, z_3\}$ and $\{z_1, z_4\}$, and let $S_2$ denote the 2-change replacing $\{z_2, z_5\}$ and $\{z_1, z_4\}$ with $\{z_1, z_5\}$ and $\{z_2, z_5\}$. From Fig. 3, it is evident that we can treat $\Delta _1$ and $\eta = \Vert z_2 - z_5\Vert - \Vert z_1 - z_5\Vert$ as independent variables, as long as we condition on $R = r$.

##### Lemma 24

Let $\Delta ^{\textrm{link}}_\textrm{min}$ denote the minimum improvement of any type 1b pair of 2-changes, and assume that $\mathcal {X}\subseteq [-D, D]^d$. Then

$$\begin{aligned} \mathbb {P}(\Delta ^{\textrm{link}}_\textrm{min} \in (0,\epsilon ]) = O\left( \frac{n^5 d^{3/4}D^{3/2}}{\sigma ^{3}} \epsilon ^{3/2} \right) . \end{aligned}$$

##### Proof

The proof follows along the exact same lines as Lemma 23. small modifications. $\square$

Lemmas 20, 23 and 24 enable us to prove an upper bound to the smoothed complexity of 2-opt in the present probabilistic model.

##### Theorem 25

The expected number of iterations performed by 2-opt for smoothed Euclidean instances of TSP in $d \ge 2$ dimensions is bounded from above by $O\left( d D^2 n^{4 + \frac{1}{3}}/\sigma ^2 \right)$.

##### Proof

We assume for this proof that the entire instance is contained within $[-D, D]^d$, with $D = \Theta (1 + \sigma \sqrt{n \log n})$. This occurs with probability at least $1 - 1/n!$. Thus, with probability at least $1 - 1/n!$, the longest tour in the instance has length at most $2\sqrt{d}Dn$. The assumption that the entire instance lies within this hypercube enables us to use Lemmas 20, 23 and 24, which were proved under this assumption.

Let *E* denote the event that, among all type 0 and type 1 linked pairs of 2-changes, the pair with the smallest improvement is of type 0, and let $E^c$ denote the event that this pair is of type 1a or type 1b. Let the random variable *T* denote the number of iterations taken by 2-opt to reach a local optimum.

We first compute $\mathbb {E}(T|E)$. We apply Lemma 1 with $\alpha = 2$, which is feasible due to Lemma 20. We then obtain immediately that $\mathbb {E}(T|E) = O(dD^2n^4/\sigma ^2)$.

Next, we compute $\mathbb {E}(T|E^c)$. In this case, we apply Lemma 1 with $\alpha = 3/2$ (cf. Lemmas 23 and 24). This yields $\mathbb {E}(T|E^c) = O(dD^2n^{4+\frac{1}{3}}/\sigma ^2)$.

Combining the bounds for *E* and $E^c$ yields the result. $\square$

## Improving the Analysis for $d \ge 3$

The bottleneck in Theorem 25 stems from Lemmas 23 and 24, which bound the probability that any linked pair of type 1a or type 1b improves the tour by at most $\epsilon$. The probability given by these lemmas is proportional to $\epsilon ^{3/2}$, which yields an extra factor of $n^{1/3}$ compared to type 0 linked pairs.

For $d \ge 3$, we can improve this to $\epsilon ^2$, yielding improved smoothed complexity bounds. The key to this improvement is to use the second part of Corollary 6 to bound the density of $\eta _i$ as in Lemma 12. This immediately yields the following result on $\eta _i = \Vert a - z_i\Vert - \Vert b - z_i\Vert$.

### Lemma 26

Let $i \in [2]$, and assume that $\mathcal {X}\subseteq [-D, D]^d$. The density of $\eta _i$ in $d \ge 3$ dimensions, conditioned on $A_i = a_i$ and $R = r$, is bounded from above by

$$\begin{aligned} O\left( \frac{a_i + r}{a_i r} \cdot \left( \sqrt{d} + \frac{D\min \{r, a_i\}}{\sigma ^2}\right) \right) . \end{aligned}$$

### Proof

We call the desired density $f_{\eta _i|A=a_i,R=r}$. From Lemma 12, we know that

$$\begin{aligned} f_{\eta _i|A_i=a_i,R=r}(\eta ) \le \frac{a_i+r}{a_i r} \cdot \frac{f_{\phi _i|A_i=a_i,R=r}(\phi _i(\eta ))}{|\sin \phi _i(\eta )|}. \end{aligned}$$

Since $d \ge 3$, we can use the second part of Corollary 6 to obtain the desired bound, making use of the assumption that all points fall within $[-D,D]^d$. $\square$

Lemma 26 enables us to find an improved version of Lemma 15.

### Lemma 27

Let $\Delta$ denote the improvement of a 2-change in $d \ge 3$ dimensions. Let $i \in [2]$, and assume that $\mathcal {X}\subseteq [-D, D]^d$. Then

$$\begin{aligned} \mathbb {P}(\Delta \in (0, \epsilon ] |A_i = a_i, R = r) = O\left( \left( \frac{\sqrt{d}}{\min \{a_i, r\}} + \frac{D}{\sigma ^2}\right) \cdot \epsilon \right) . \end{aligned}$$

### Proof

Let $j = 3 - i$. We assume that $\eta _j = t$ is fixed by the adversary. Then $\Delta \in (0, \epsilon ]$ iff $\eta _i \in (-t, -t + \epsilon ] =: I$, an interval of size $\epsilon$. By Lemma 26, we have a bound for the density of $\eta _i$. Thus, we find

$$\begin{aligned} \mathbb {P}(\Delta \in (0, \epsilon ] |A_i = a_i, R=r) = O\left( \frac{a_i + r}{a_i r} \cdot \left( \sqrt{d} + D\min \{r, a_i\}/\sigma ^2\right) \cdot \epsilon \right) . \end{aligned}$$

Considering the cases $a_i \le r$ and $a_i < r$ separately and using the assumptions that all points lie within $[-D, D]^d$ and that $D \ge 1$ and $\sigma \le 1$ yields the stated result. $\square$

The following lemma now yields the probability that any linked pair of 2-changes improves the tour by at most $\epsilon$. We omit the proof, since it follow easily from Lemma 27 along the same lines as the lemmas in Sect. 4.

### Lemma 28

Let $\Delta ^{\textrm{link}}_\textrm{min}$ denote the minimum improvement of any linked pair of 2-changes of type 0 or type 1 for $d \ge 3$, and assume that $\mathcal {X}\subseteq [-D, D]^d$. Then

$$\begin{aligned} \mathbb {P}(\Delta ^\textrm{link}_{\textrm{min}} \in (0, \epsilon ]) = O\left( \frac{D^2 n^6 \epsilon ^2}{\sigma ^4} \right) . \end{aligned}$$

We then obtain our result for $d \ge 3$.

### Theorem 29

The expected number of iterations performed by 2-opt for smoothed Euclidean instances of TSP in $d \ge 3$ dimensions is bounded from above by $O\left( \sqrt{d}D^2 n^4/\sigma ^2 \right)$.

### Proof

The theorem follows immediately from applying Lemmas 1 and 28, since by Lemma 2 any tour in our smoothed instance has length at most $2\sqrt{d}Dn$ with probability at least $1 - 1/n!$. $\square$

## Discussion

**Table 1:** Previous and current smoothed complexity bounds for Gaussian noise, for $\sigma = O(1/\sqrt{n\log n})$

	Englert, Röglin and Vöcking [8]	Manthey and Veenstra [13]	This paper
$d = 2$	$O\left( n^{4+\frac{1}{3}}/\sigma ^{5+\frac{1}{3}}\cdot \log \frac{n}{\sigma }\right)$	–	$O\left( n^{4+\frac{1}{3}}/\sigma ^2\right)$
$d = 3$	$O\left( n^{4+\frac{1}{3}}/\sigma ^8 \cdot \log \frac{n}{\sigma }\right)$	–	$O\left( n^4/\sigma ^2 \right)$
$d \ge 4$	$O\left( c_d \cdot n^{4+\frac{1}{3}}/\sigma ^{8d/3}\right)$	$O\left( \sqrt{d}n^4/\sigma ^4 \right)$	$O\left( \sqrt{d}n^4/\sigma ^2\right)$

Note that for $d \ge 4$, the bounds of Englert et al. include a factor $c_d$ which is super-exponential in *d*

**Table 2:** Previous and current smoothed complexity bounds for Gaussian noise, for $\sigma = \Omega (1/\sqrt{n\log n})$

	Englert, Röglin and Vöcking [8]	Manthey and Veenstra [13]	This paper
$d = 2$	$O\left( n^7 \log ^{3+\frac{2}{3}} n \right)$	–	$O\left( n^{5+\frac{1}{3}}\log n\right)$
$d = 3$	$O\left( n^{8 + \frac{1}{3}}\log ^5 n \right)$	–	$O\left( n^5\log n \right)$
$d \ge 4$	$O\left( c_d \cdot n^{4+\frac{1 + 4d}{3}} \log ^{1 + \frac{4d}{3}}n\right)$	$O\left( \sqrt{d}n^6\log ^2 n \right)$	$O\left( \sqrt{d}n^{5} \log n\right)$

Note that for $d \ge 4$, the bounds of Englert et al. include a factor $c_d$ which is super-exponential in *d*

For convenience, we provide comparisons of the previous smoothed complexity bounds with our bound from Theorem 25 in Tables 1 and 2. These bounds are provided both for small values of $\sigma$ and for large values, meaning $\sigma = \Omega (1/\sqrt{n\log n})$ and $\sigma = O(1/\sqrt{n \log n})$.

Observe from Tables 1 and 2 that the bound for $d = 2$ has a worse dependence on *n* compared to the bound for $d \ge 3$. The technical reasons for this difference can be understood from Sect. 5. A more intuitive explanation for the difference is that our analysis benefits from large angles between edges in the smoothed TSP instance. In $d = 2$, the density of these angles is maximal when they are small, while for $d \ge 3$ it is maximal when the angles are large. In effect, this means that the adversary has less power to specify these angles to our detriment when $d \ge 3$.

From these tables, the greatest improvement is made for $d = 3$, where we improve by $n^{3+\frac{1}{3}}\log ^{4}n$ in the large $\sigma$ case, and by $\root 3 \of {n}\log (n/\sigma )/\sigma ^6$ for small $\sigma$. For $d = 2$, the improvement is more modest at $n^{1+\frac{2}{3}}\log ^{2+\frac{2}{3}}n$ for large $\sigma$ and $\log (n/\sigma )/\sigma ^{3 + \frac{1}{3}}$ for small $\sigma$. For $d \ge 4$, we improve by $n\log n$ for large $\sigma$, and by $\sigma ^{-2}$ for small $\sigma$.

Note that we improve upon previous bounds mainly in the dependence on the perturbation strength. In an intuitive sense, this is most substantial for instances that are weakly perturbed from the adversarial instance, or in other words, that are close to worst case. In addition, the small-$\sigma$ case is considered more interesting for a smoothed analysis, since small $\sigma$ model the intuition of smoothed analysis of a small perturbation, while large $\sigma$ reduce the analysis basically to an average-case analysis In order to improve the explicit dependence on *n*, which is the same as for Manthey and Veenstra [13], we believe new techniques are necessary.

As a final comment, we note that the techniques we employed in Sects. 3 and 5 can also be used to improve and significantly simplify the analysis of the one-step model used by Englert et al. [8]. For $d \ge 3$, the improvement amounts to a factor of $n^{1/3}\phi ^{1/6}\log (n\phi )$, while for $d = 2$, the improvement is just $\log (n\phi )$, where $\phi$ denotes the upper bound of the density functions used in the one-step model.

## Footnotes

1 This assumption is only valid for $d = 2$. To see this, observe that by conditioning on $A_i = a_i$, the point $z_i$ is distributed uniformly on the $(d-1)$-sphere with radius $a_i$. For $d > 2$, the density of $\phi$ is thus concentrated near $\phi = \pi /2$. An upper bound for this density can be obtained by setting $s = 0$ in Theorem 5, yielding $O(\sqrt{d})$. As Englert et al. assume *d* to be constant, this has no effect on their eventual result.
